# Detecting RNA-protein proximity at DNA double-strand breaks using combined fluorescence *in situ* hybridization with proximity ligation assay

**DOI:** 10.1016/j.xpro.2023.102096

**Published:** 2023-02-03

**Authors:** Adele Alagia, Ruth F. Ketley, Monika Gullerova

**Affiliations:** 1 Sir William Dunn School of Pathology, University of Oxford, South Parks Road, Oxford OX1 3RE, UK

**Keywords:** Cell Biology, Microscopy, Molecular Biology, In Situ Hybridization, Molecular/Chemical Probes

## Abstract

RNA transcribed at DNA double-strand breaks (DSBs) contributes to accurate DNA repair. Here, using the repair factors 53BP1 and TIRR as examples, we combine the fluorescence *in situ* hybridization (FISH) and proximity ligation assay (PLA) techniques to determine protein proximity to DSB-transcribed RNA. In this FISH-PLA protocol, we detail steps for designing DNA probes and image analysis using CellProfiler™ software. This approach has many potential applications for the study of the RNA-binding proteins and nascent RNA interactions.

For complete details on the use and execution of this protocol, please refer to Ketley et al. (2022).^1^

## Highlights

• Induce sequence-specific double-strand breaks (DSBs) using U2OS-A*si*SI-ER cells
• Target *de novo* transcribed RNA produced at DSBs with specifically designed FISH-PLA probes
• Detect the close proximity of a protein of interest to DSB-derived RNA
• Detect and quantify FISH-PLA signals using confocal microscopy and CellProfiler

## Abstract (editor highlights)

Publisher’s note: Undertaking any experimental protocol requires adherence to local institutional guidelines for laboratory safety and ethics.

## Graphical abstract

**Figure:**
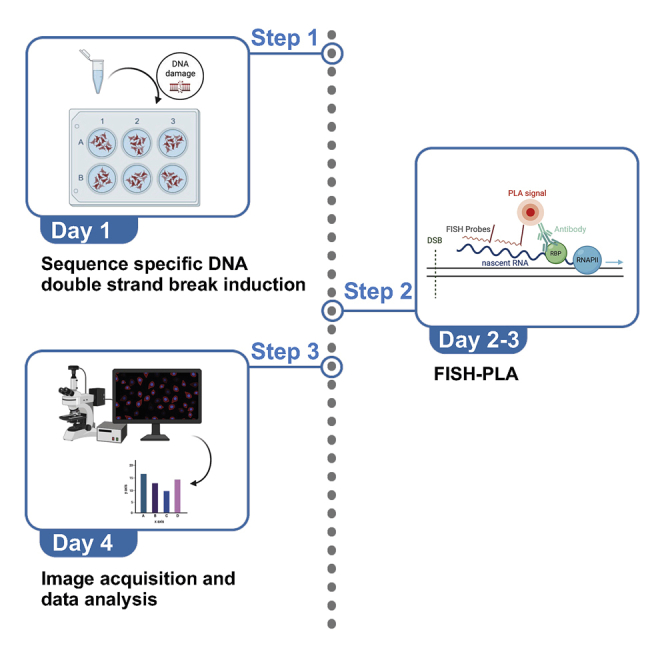

## Before you begin

1. Design and order DNA FISH probes.
2. Prepare diethyl pyrocarbonate (DEPC) treated H_2_O.
3. Prepare buffers.
4. Culture cells, maintaining them in a transcriptionally active state (<70%–80% confluency).

**Figure 1:**
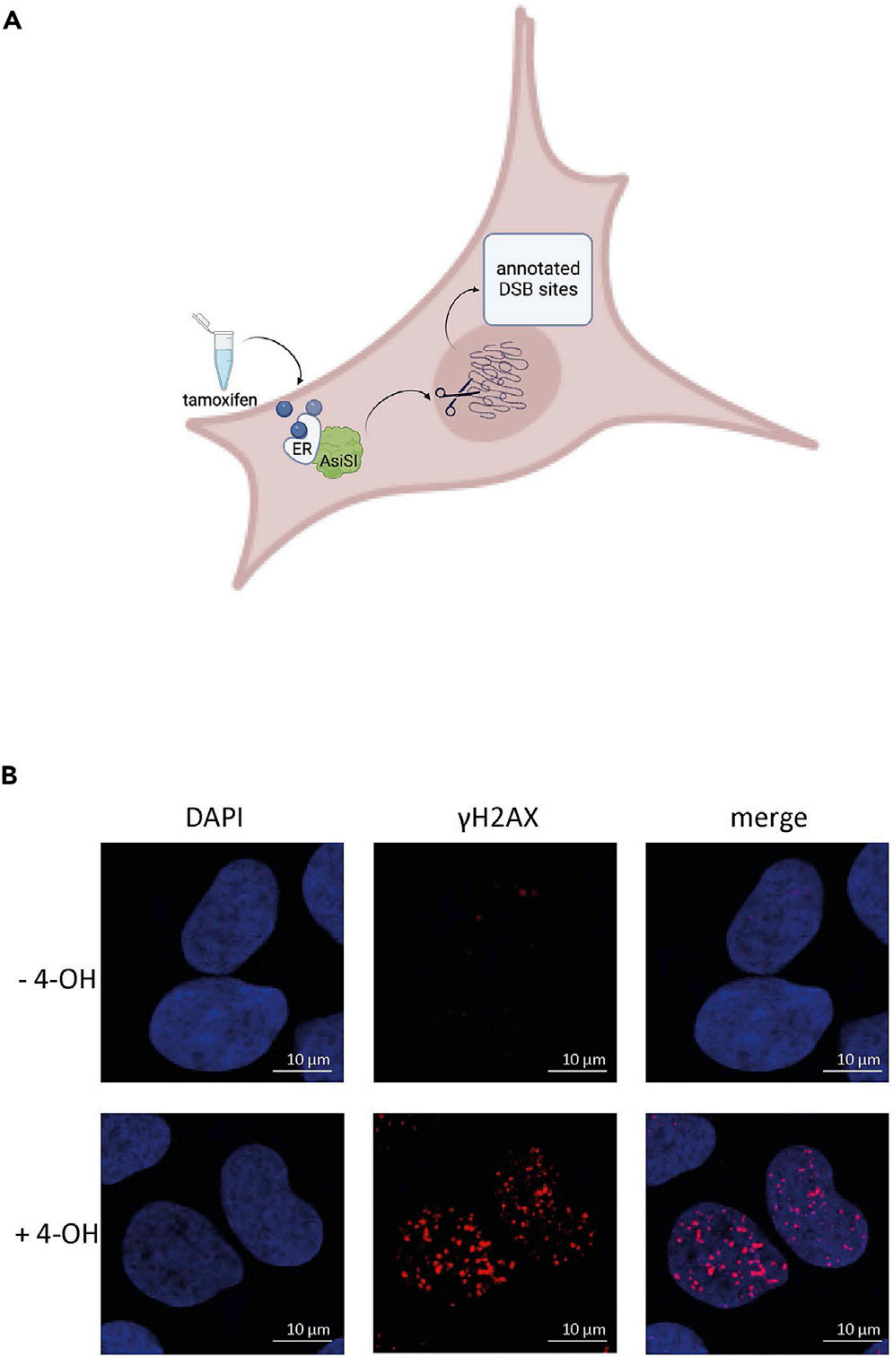
Sequence-specific DSBs induction in the U2OS-A*si*SI-ER cell line (A) Diagram summarizing the strategy for sequence specific DSBs induction. (B) Immunofluorescence images showing γH2AX foci induced by 4-hydroxytamoxifen in U2OS-A*si*SI-ER cell line.^1^

Here, we described a modified PLA protocol using DNA oligonucleotide probes and a primary antibody against a protein of interest (POI) to detect proximity of *de novo* transcribed RNA and the POI upon DSB induction in the U2OS-A*si*SI-ER cell line. The A*si*SI restriction enzyme, linked to a modified ligand-binding domain of the estrogen receptor (ER), which translocates to the nucleus after the addition of 4-hydroxytamoxifen (4-OHT), allows the site/sequence-specific induction of the DSB, as measured by γH2AX levels^1^^,^^2^ (Figures 1A and 1B).

### Preparation of reagents

**Timing: approximately 1 day**

5. DEPC-H_2_O: Add 1 mL of DEPC to 1,000 mL of ddH_2_O in a screw-cap glass bottle. Incubate for 12–18 h at 20°C in a fume hood with swirling. Autoclave to inactivate DEPC. Store at 20°C.
6. RNAse-free buffers: prepare buffers using the autoclaved DEPC-H_2_O. Buffer recipes are shown in the materials and equipment section.

***Note:*** In the event that solutions are not autoclavable (i.e., Sucrose), sterile filter the solution using a 0.22 μm cellulose nitrate or cellulose acetate membrane.

**Figure 2:**
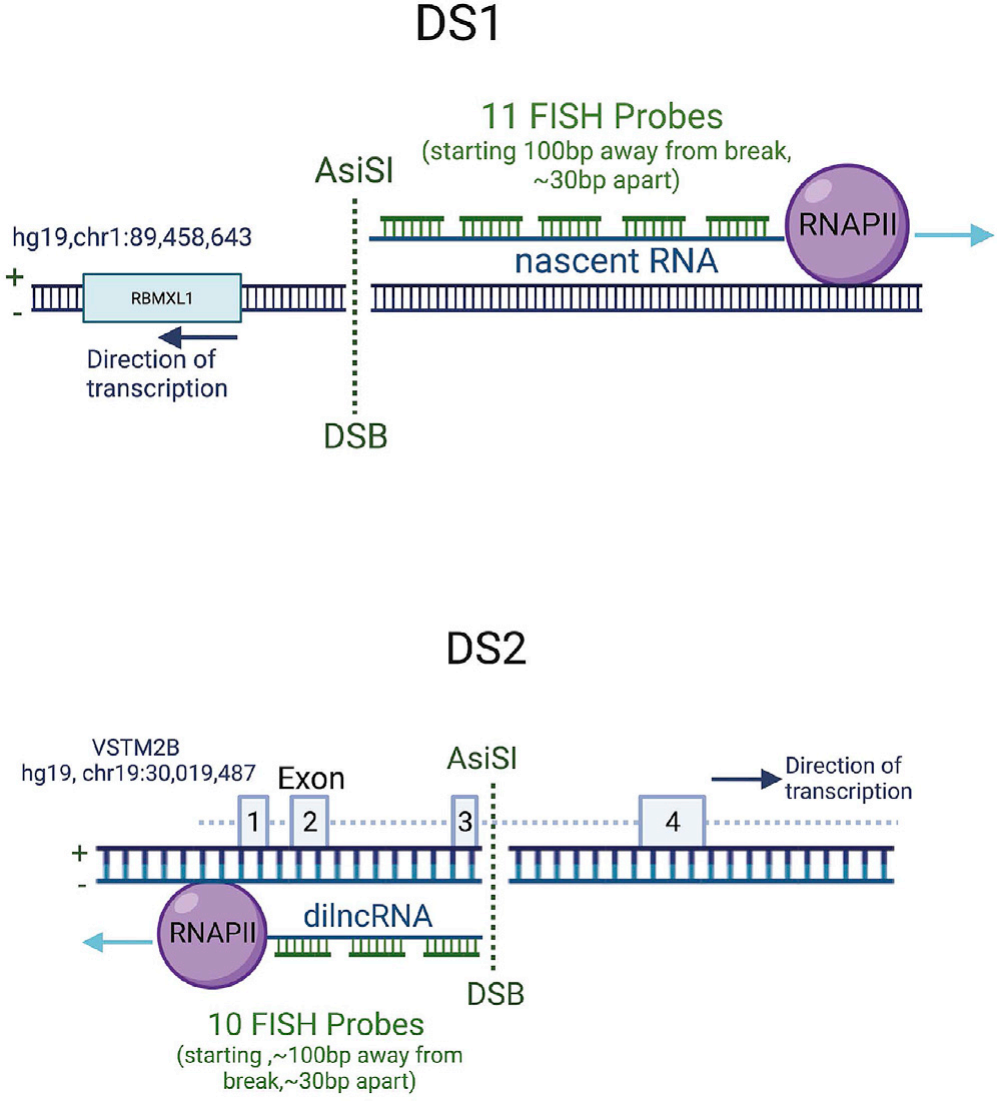
Design strategy of the DNA probes at DS1 (RBMXL1) and DS2 (VSTM2B) loci DNA probes (in green) hybridize to the antisense RNA *de novo* transcribed by RNA polymerase II at double strand breaks.^1^
  b. The first DNA probe should hybridize ∼100 nucleotides away from the A*si*Si cut site, and the probes should span around 1kb in this region. The DNA probe set should have a complementary sequence to the *de novo* antisense RNA transcript. Each DNA probe should be ∼50nt long and spaced more than 30 bases apart to allow adequate space for PLA enzymatic reactions. To ensure comparable hybridization efficiency, each DNA probe should have the same GC content (∼55%). Check that the DNA probes have uniqueness against the genome with a BLAT search.
  c. Add the following to the DNA complementary sequence: 41 Deoxy-adenosine bases and the 18 nucleotide sequence TAT GAC AGA ACT AGA CAC.

7. Design of the DNA probes:
  a. Import the DNA sequence of the gene containing the A*si*SI cut site (e.g., hg19, chr1:89,458,643 RBMXL1/CCBL2 (DS1) or hg19, chr19:30,019,487 VSTM2B (DS2)) into the SnapGene software and identify the A*si*SI Restriction Enzyme Cut Site (GCGAT/CGC) (Figure 2).

The sequences of the DNA probes used in this study are shown in Table 1.

8. Optimization of the primary antibody: Antibody choice is critical to the success of the FISH-PLA assay. Generally, antibodies applicable for immunofluorescence experiments should work in the FISH-PLA setting. The working concentration of the primary antibody should be set starting from manufacturer’s recommended concentration and titrated at different dilutions to establish specific signals *vs* background. The optimal primary antibody concentration could also be determined in a canonical dual antibody PLA assay.

**Table 1:** Oligonucleotides used in FISH-PLA experiments

Probe name	Sequence (5′>3′)
FISH_PLA _DS1_1	CCGGCCGACTCCACAGCCAATGGAGTTCCCTGAGTCCACACCCATAGCC AAAAA AAAAAAAAAAAAAAAAAAAAAAAAAAAAAAAAAAATATGACAGA ACTAGACAC
FISH_PLA _DS1_2	CCAAATTCCATACCCATATCCAAGTCCCCGCCCCACTAACTCGAGCCCG CAAAAA AAAAAAAAAAAAAAAAAAAAAAAAAAAAAAAAAATATGACAGA ACTAGACAC
FISH_PLA _DS1_3	GCCCATCCTTGCAAACCAGTCCTCGTCCTATAACCTGCCCCAACCCGAT CAAAAA AAAAAAAAAAAAAAAAAAAAAAAAAAAAAAAAAATATGACAG AACTAGACAC
FISH_PLA _DS1_4	CCCTGCCCTCTCTCAGGCACCTTCCAAAGTTATTCCAACCCGATCCCTCC AAAAA AAAAAAAAAAAAAAAAAAAAAAAAAAAAAAAAAATATGACAGA ACTAGACAC
FISH_PLA _DS1_5	GTTCCTTTACGCTTTTTAACCCCACACCCCCATCTCAAATGACAAATGCA AAAAA AAAAAAAAAAAAAAAAAAAAAAAAAAAAAAAAAATATGACAGA ACTAGACAC
FISH_PLA _DS1_6	GTCATTTTTCTTCCTTCTTCAGTCTCACATGTCCCTTTCATTCTTAGTTTAA AAAAA AAAAAAAAAAAAAAAAAAAAAAAAAAAAAAAATATGACAGAAC TAGACAC
FISH_PLA _DS1_7	TTCGCTGACACTGCCCTCCCCAAAGAAGTCACCAGTGGTTTCCTTGTTTT AAAAA AAAAAAAAAAAAAAAAAAAAAAAAAAAAAAAAAATATGACAGA ACTAGACAC
FISH_PLA _DS1_8	CCCTCTAGCCTCTCATCCTCTTAAAATAGAAGAGGAAAAATATCCAAACA AAAA AAAAAAAAAAAAAAAAAAAAAAAAAAAAAAAAAAATATGACAGAA CTAGACAC
FISH_PLA _DS1_9	GAAGAGTATATTCCTTTAATCAATGTATACTTTTGTTCCCTCCTTTAGAAA AAAAA AAAAAAAAAAAAAAAAAAAAAAAAAAAAAAAAATATGACAGAA CTAGACAC
FISH_PLA _DS1_10	CACATCCAGGCTCGCCCCTACCTGTACACAATAATTTCTCCATGCACC TAAAAAA AAAAAAAAAAAAAAAAAAAAAAAAAAAAAAAAAATATGACA GAACTAGACAC
FISH_PLA _DS1_11	TGAGATGTAGTATGATCACCAATCGCGGGGGACCCCACAGCCAGTG CGCGAAAA AAAAAAAAAAAAAAAAAAAAAAAAAAAAAAAAAAATAT GACAGAACTAGACAC
DS2_VSTM2B_FISH_1 F	AGGATGCAACTAAAATCAGCGTAAGTGTGGAGCCCAGCGCGGGCC GCGGGAAAA AAAAAAAAAAAAAAAAAAAAAAAAAAAAAAAAAAAATATGACAGA ACTAGACAC
DS2_VSTM2B_FISH_2 F	CCTTGCCTAAAGGCGGATCCGAGTTCCCCTAGCCAGAAGGCCGCG AGCCTAAAA AAAAAAAAAAAAAAAAAAAAAAAAAAAAAAAAAAAAT ATGACAGAACTAGACAC
DS2_VSTM2B_FISH_3 F	CTGGGAAACTCTTGGAAAGCCGGACGTCCTTTGTGCCCTCAACCC CCATCAAAAA A AAAAAAAAAAAAAAAAAAAAAAAAAAAAAAAAAATATGACAGAACT AGACAC
DS2_VSTM2B_FISH_4 F	CATCCAGTCTCCCCAGTTCAGCCGGCAGGTGTGCACCAGGGCAGCC ACCCAAAA AAAAAAAAAAAAAAAAAAAAAAAAAAAAAAAAAAAATAT GACAGAACTAGACAC
DS2_VSTM2B_FISH_5 F	GTCCAGACACAGCGGCCCTCCCTCCAGTTCTTCACGGCTCCCAGTG AGCTAAAAA AAAAAAAAAAAAAAAAAAAAAAAAAAAAAAAAAAATA TGACAGAACTAGACAC
DS2_VSTM2B_FISH_6 F	GTCTGCCTCAAAACCCACACTCTCTTACACACTCTAGTGTGCTCGC GTGCAAAAA AAAAAAAAAAAAAAAAAAAAAAAAAAAAAAAAAAAT ATGACAGAACTAGACAC
DS2_VSTM2B_FISH_7 F	GCCACCAGAAAACCGAGTACCGGAAAGCCGGCAGGACCTCCAGC CCTCAGAAAA AAAAAAAAAAAAAAAAAAAAAAAAAAAAAAAAAAA ATATGACAGAACTAGACAC
DS2_VSTM2B_FISH_8 F	TGCTCGCTGGCTGCGGCCCTCTGATGGGTGCTGGGTAGTGAAGGA AGCCCAAAA AAAAAAAAAAAAAAAAAAAAAAAAAAAAAAAAAAAA TATGACAGAACTAGACAC
DS2_VSTM2B_FISH_9 F	CAGCAGTGGTTACCCCCAAACCTGTCAATTATTTTGACAGCATCGCT GTTAAAAA AAAAAAAAAAAAAAAAAAAAAAAAAAAAAAAAAAATAT GACAGAACTAGACAC
DS2_VSTM2B_FISH10 F	AGGGAGGGAAGCAGCGGGCTTCACTCGCGCAGGGCGCCGCCCT GTGGCGCAAAA AAAAAAAAAAAAAAAAAAAAAAAAAAAAAAAAA AAATATGACAGAACTAGACAC

## Key resources table

**Table:** 

REAGENT or RESOURCE	SOURCE	IDENTIFIER
**Antibodies**		

53BP1 Anti-Rabbit	Novus Biologicals	NB100-304
TIRR Anti-Rabbit	Novus Biologicals	NBP1-92209
γH2AX Anti-Rabbit	Abcam	Ab11174

**Chemicals, peptides, and recombinant proteins**		

Dulbecco’s Modified Eagle Medium (DMEM)	Gibco	11965092
Fetal bovine serum (FBS)	Sigma-Aldrich	F9665
Penicillin/Streptomycin (Pen/Strep)	Gibco	10378016
(Z)-4-hydroxy tamoxifen (4-OHT)	Cayman	14854
α-Amanitin	Cayman	17898
DEPC (diethyl pyrocarbonate)	Sigma-Aldrich	D5758
PIPES	Sigma-Aldrich	P3768
Sucrose	Sigma-Aldrich	S0389
NaCl	Sigma-Aldrich	S9888
MgCl_2_	Sigma-Aldrich	63068
EGTA	Sigma-Aldrich	E3889
Triton X-100	Sigma-Aldrich	T8787
SSC 20×	Gibco	15557044
Bovine serum albumin fraction V (BSA)	Sigma-Aldrich	10735086001
ytRNA	Invitrogen	AM7119
Sheared salmon sperm DNA (sssDNA)	Invitrogen	15632011
RNAsin PLUS	Promega	N2611
Poly-l-lysine	Sigma-Aldrich	P8920
PBS	Sigma-Aldrich	D8537
PFA 4% in PBS	Alfa Aesar	J61899
Duolink In Situ PLA Probe-Anti-Rabbit MINUS	Sigma-Aldrich	DUO92006-100RXN
Duolink In Situ Wash A	Sigma-Aldrich	DUO82046-1EA
Duolink In Situ Wash B	Sigma-Aldrich	DUO82048-1EA
Duolink In Situ Mounting Media with DAPI	Sigma-Aldrich	DUO82040-5ML
Duolink In Situ Detection Reagents Red	Sigma-Aldrich	DUO92008-100RXN

**Experimental models: Cell lines**		

U2OS-A*si*SI-ER	Massip et al.^2^	N/A

**Oligonucleotides**		

DNA probes (Please see Table 1)	Sigma-Aldrich	N/A

**Software and algorithms**		

SnapGene	Insightful Science	RRID: SCR_015052
ImageJ	National Institute of Health	RRID: SCR_003070
CellProfiler	Broad Institute of MIT and Harvard	RRID: SCR_007358
GraphPad Prism 9.4.1	GraphPad Software Inc	RRID: SCR_002798

**Other**		

6-well plate	Corning	3516
20-mm coverslips	Fisher Scientific	12323138
Microscope slides	Fisher Scientific	12373118
Olympus Fluoview FV1200	Olympus	RRID: SCR_017564

## Materials and equipment

Basic laboratory materials such as a laminar culture hood, CO_2_ incubator, microcentrifuge, glassware, and plasticware have not been mentioned in the tables below but are required for the protocol.

**Table:** Pre-extraction buffer

Reagent	Final concentration	Amount
PIPES (0.5 M pH 6.9)	10 mM	200 μL
Sucrose (1 M)	300 mM	3 mL
NaCl (5 M)	100 mM	200 μL
MgCl_2_ (1 M)	3 mM	30 μL
EGTA (1 M)	3 mM	30 μL
Triton X-100 (10%)	0.7%	700 μL
DEPC-H_2_O	–	5.84 mL
**Total**	**–**	**10 mL**

Store at 4 degrees for up to one month.

**Table:** FISH-PLA blocking buffer

Reagent	Final concentration	Amount
SSC (20×)	2×	100 μL
BSA (10%)	2%	200 μL
Triton X-100 (10%)	0.5%	50 μL
ytRNA (10 μg/μL)	40 ng/μL	4 μL
sssDNA (10 μg/μL)	40 ng/μL	4 μL
DEPC-H_2_O	–	642 μL
**Total**	**–**	**1 mL**

Store at 4 degrees for up to one week.

**Table:** FISH-PLA DNA probe buffer

Reagent	Final concentration	Amount
SSC (20×)	2×	50 μL
Triton X-100 (10%)	0.5%	25 μL
ytRNA (10 μg/μL)	20 ng/μL	1 μL
sssDNA (10 μg/μL)	20 ng/μL	1 μL
RNAsin PLUS (40 U/μL)	1 U/ μL	12.5 μL
DNA probes (10 μM)	100 nM	5 μL each
DEPC-H_2_O	–	360.5 μL
**Total**	**–**	**500 μL**

Prepare fresh and keep on ice.

**Table:** SSC 2× washing buffer

Reagent	Final concentration	Amount
SSC (20×)	2×	5 mL
BSA (10%)	2%	5 mL
Triton X-100 (10%)	0.1%	5 mL
DEPC-H_2_O	–	35 mL
**Total**	**–**	**50 mL**

Store at 4 degrees for up to one week.

## Step-by-step method details

**Figure 3:**
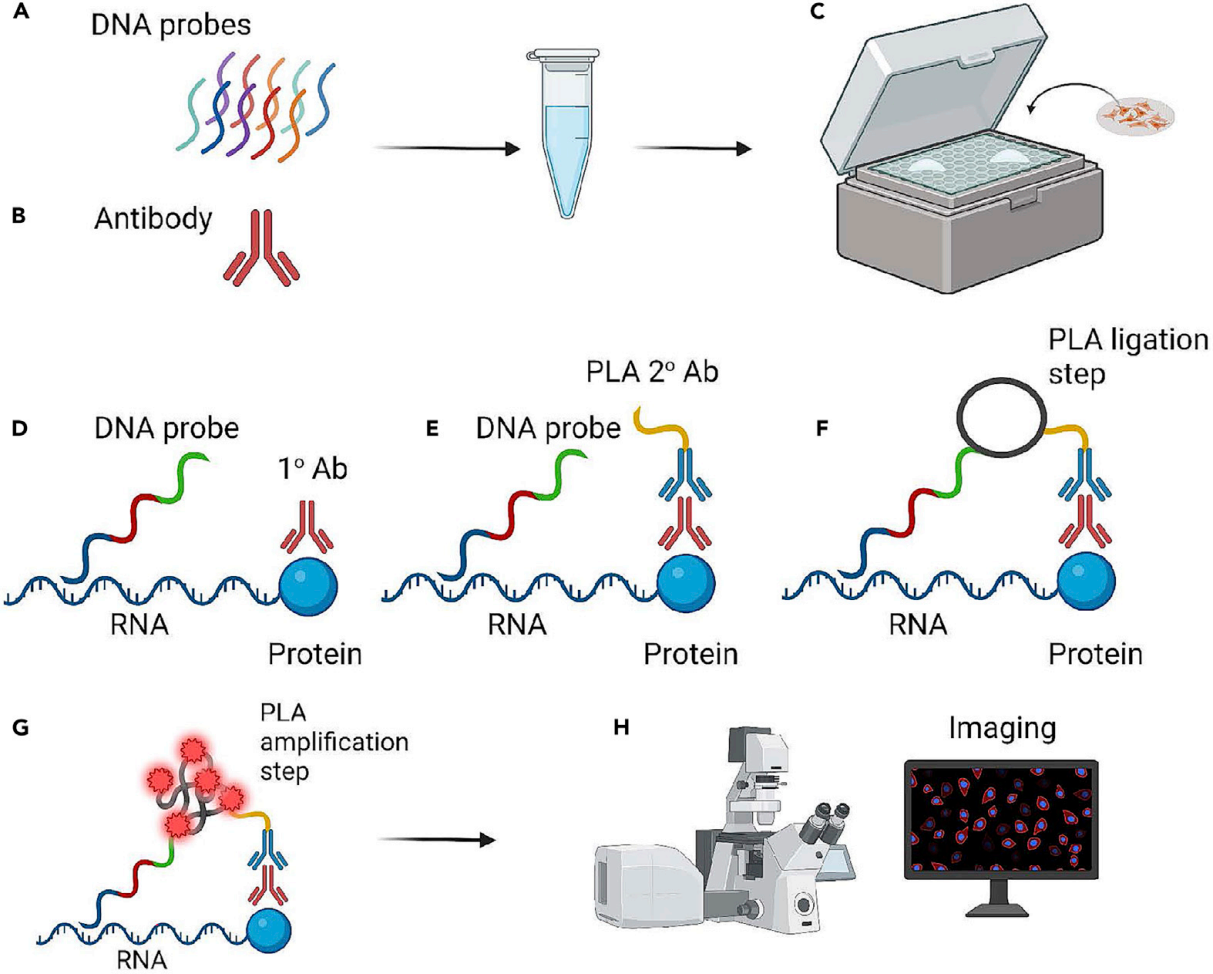
Diagram of a workflow of the FISH-PLA protocol (A) DNA probe hybridization. (B) Primary antibody incubation. (C) Illustration of the humidity chamber and the sample coverslip reaction during DNA probe hybridization, antibody incubation, and Duolink PLA steps. (D) Recognition of the target RNA by the DNA probe and the RNA binding protein by the primary antibody. (E) Incubation of the MINUS Duolink PLA probe. (F) Duolink ligation step between the PLUS DNA probe and the MINUS Duolink PLA probe. (G) Duolink Amplification step resulting in the formation of FISH-PLA foci. (H) Imaging analysis using ImageJ and CellProfiler softwares.

For an overview of the FISH-PLA workflow, see Figure 3.

### Preparation of cells

**Timing: 24 h**

The following steps describe the cell seeding procedure on poly-l-lysine coated coverslips in a 6 well plate format.

***Note:*** The use of coating reagents such as poly-l-lysine is optional. Other coating reagents could also potentially be used such as Matrigel®, gelatin, or collagen.

1. Insert one sterile coverslip (20 mm diameter) into each well of a 6 well plate.
2. Add 2 mL of 0.01% poly-L-lysine solution in PBS into each well of a 6 well plate and incubate at 37 °C for 30 min.
3. Vacuum aspirate the poly-l-lysine solution and wash twice with 2 mL PBS.
4. Seed 3 × 10^5^ U2OS-AsiSI-ER cells in 2 mL of complete DMEM (1× Pen/Strep, 10% FBS).
5. Cells should be allowed to attach on coverslips for 24 h incubating at 37°C with 5% CO_2_.

**CRITICAL:** After poly-l-lysine incubation, wells should be washed at least twice with PBS as residual poly-l-lysine is toxic and may cause cell death. To ensure a successful FISH-PLA assay, cells should be maintained in a transcriptionally active state, and therefore the confluency should reach no more than 60%–70% after 24 h.

### DSB induction, cell fixation, blocking

**Timing: 6 h**

The steps below describe DSB induction, cell fixation and blocking.

**CRITICAL:** Before starting the DSB induction, switch on the hybridization oven and a thermoblock, setting the temperature at 95 °C. Additionally, pre-warm a humidified chamber to 37°C.

6. Vacuum aspirate the culture media.
7. Add 500nM of 4-hydroxytamoxifen (4-OHT) in 2 mL of complete DMEM (1× Pen/Strep, 10% FBS) and incubate 37°C with 5% CO_2_ for 4 h.
8. Wash twice with 2 mL ice cold PBS.
9. Add 2 mL of PFA (4% in PBS) and incubate for 15 min at room temperature to fix the cells, then wash 3 times with 2 mL ice cold PBS and permeabilize with 0.2%–0.5% Triton X100 for 10 min.
10. Wash 3 times with PBS. Add 80 μL of FISH-PLA Blocking Buffer (per coverslip) to a pre-warmed humidified chamber and place the coverslip face down on the FISH-PLA blocking solution. Incubate for 1 h at 37°C.

### FISH-PLA DNA probes buffer preparation and oligonucleotide hybridization

**Timing: 16–18 h**

The following steps summarize FISH-PLA probes hybridization, washing steps and primary antibody incubation.

11. FISH-PLA DNA probe buffer should be prepared in two steps.
  a. Firstly, add SSC, diluted DNA probes, and DEPC-H_2_O to an RNAse-free 1.5 mL tube. Incubate at 95°C for 3 min followed by immediate snap-cooling on ice.
  b. After cooling, add Triton X-100, RNAsin plus, and sssDNA, keeping the solution on ice.

**CRITICAL:** The denaturation and snap-cooling steps are important to preserve the linearization of the DNA probes and avoid the formation of DNA secondary structure.

12. Add 60 μL of FISH-PLA probe buffer onto glass slides and place them in the humidified chamber. Incubate in a hybridization oven at 95°C for 3 min, followed by 12–18 h incubation at 37°C.

### FISH-PLA DNA probe washing

**Timing: 30 min**

13. Take the coverslip from the humidified chamber and place into new 6 well plate. Add 2 mL of 2× SSC washing buffer and incubate at 20°C for 5 min with gentle rocking. Repeat the SCC washing step twice more.
14. Wash twice with 2 mL PBS for 5 min, gently rocking.

### PLA blocking and primary antibody incubation

**Timing: 16–18 h**

15. Add 80 μL of Duolink Blocking Solution to each coverslip and incubate at 37°C for 1 h.
16. Dilute the primary antibody (see key resources table) in Duolink Antibody Diluent and add 60 μL of the primary antibody solution to each coverslip. Incubate 12–18 h at 4°C in a humidified chamber.

**CRITICAL:** Do not allow sample to dry out. To remove the excess buffer, quickly tap the coverslip on a tissue.

### PLA Duolink protocol

**Timing: 4 h**

The steps herein describe PLA part of the protocol using Duolink KIT components.

***Note:*** Prewarm PLA wash buffers A and B to room temperature in a water bath set at 37°C.

17. Add 2 mL of Wash Buffer A in each well of a 6 well plate, tap off the excess primary antibody solution from the coverslips and place the coverslips cell side up in the wash buffer. Incubate at 20°C for 5 min with gentle rocking. Repeat this step twice.
18. Dilute the Minus PLA probe 1:5 in Duolink Antibody Diluent.
19. Add 60 μL of the probe solution onto the parafilm in the humidity chamber.
20. Tap off the excess buffer from the coverslips and place coverslips cell side down onto the probe solution in the humidity chamber and incubate at 37°C for 1 h.

***Note:*** Thaw the Duolink ligation buffer during the minus PLA probe incubation. The buffer contains DTT which can precipitate. Resuspend the buffer by pipetting to dissolve any precipitates.

21. Add 2 mL of Wash Buffer A to each well of a 6 well plate, tap off any excess probe solution and place the coverslips cell side up in the wash buffer. Incubate at 20°C for 5 min with gentle rocking. Repeat this step twice.
22. Dilute the Duolink ligase buffer at a 1:5 ratio in DEPC-H_2_O.
23. For 60 μL of ligase reaction solution, add 1.5 μL of ligase enzyme to 58.5 μL of the diluted Duolink ligase buffer.

***Note:*** Prepare the ligation reaction solution during the washing step.

24. Add 60 μL of ligase buffer onto the parafilm in the humidity chamber.
25. Tap off the excess buffer from the coverslips and place cell side down in the humidity chamber, incubating at 37°C for 30 min.

***Note:*** Thaw the Duolink amplification buffer during the ligation reaction incubation.

26. Add 2 mL of Wash Buffer A to each well of 6 well plate, tap off the excess ligase reaction solution and place the coverslips cell side up. Incubate at 20°C for 5 min with gentle rocking. Repeat this step twice.
27. Dilute the Duolink amplification buffer at a 1:5 ratio in DEPC-H_2_O and protect from light.
28. For a 60 μL ligase reaction, add 0.75 μL of polymerase enzyme to 59.25 μL of the diluted Duolink ligase buffer.

***Note:*** prepare the amplification reaction solution during the washing step and protect from light.

29. Add 60 μL of the amplification solution onto the parafilm in the humidity chamber.
30. Tap off the excess buffer from the coverslips, place coverslips cell side down in the humidity chamber, and incubate at 37°C for 1 h and 40 min (protecting from light).
31. Add 2 mL of Wash Buffer B in each well of a 6 well plate, tap off the excess probe solution and place the coverslips cell side up. Incubate at 20°C for 10 min with gentle rocking. Repeat this step twice, protecting from light.
32. Perform the last wash with 0.01× washing buffer B at 20°C for 1 min, protecting from light.
33. Add 50 μL of Duolink *in situ* Mounting medium with DAPI to a microscope slide.
34. Tap off the excess of washing solution and mount the coverslip on the microscope slide, sealing the edges of the coverslip with clear nail varnish.

***Note:*** avoid producing air bubbles during the coverslip mounting.

**CRITICAL:** Wait 15 min before the acquisition of images at the microscope.

**Pause point:** The slides can be stored at 4°C in the dark. However, to ensure good image quality, it is recommended to image the slides within 3–4 days after mounting.

### Confocal microscope setup

**Timing: variable, mainly depends on the slide number and microscope-specific acquisition parameters (2–6 h)**

The steps below describe image acquisition and analysis using an Olympus Confocal Microscope and CellProfiler software.

**CRITICAL:** Sample slides should be imaged maintaining constant microscope parameters (e.g., laser power).

All imaging was performed using an Olympus Confocal Microscope FV1200, with a 60× objective. The filters DAPI (405nm) and Texas Red (559nm) were used, with speed settings at fast and an area of 1024 × 1024. The images were acquired using Z stacks at a 1 μm step size.

### FISH-PLA image analysis using Fiji Image J and CellProfiler software

**Timing: variable from 2–6 h**

**CRITICAL:** Confocal images acquired by the confocal microscope can be saved in oib format (Olympus Image Binary) and should be processed using Fiji Image J software.

35. Open the image in Fiji Image J software. In the options for “Bio-formats import options”, tick the box “Split channels”. Two windows corresponding to the DAPI signal (e.g., sample_name.oib – C = 0) and FISH-PLA signal (e.g., sample_name.oib – C = 1) will open.

***Note:*** Split images can be visualized in greyscale or in arbitrary colours that can be set by selecting from the main menu ‘Image/Colour/Channels tool’.

36. In the main menu of ImageJ, select Image/Stacks/Z Project to stack all the z stacks acquired at the microscope. In the window ‘ZProjection’ insert values for “Start slice” and “Stop slice” and select Max intensity from the from the drop-down menu “Projection Type”. Save the processed images as TIFF files.

**Figure 4:**
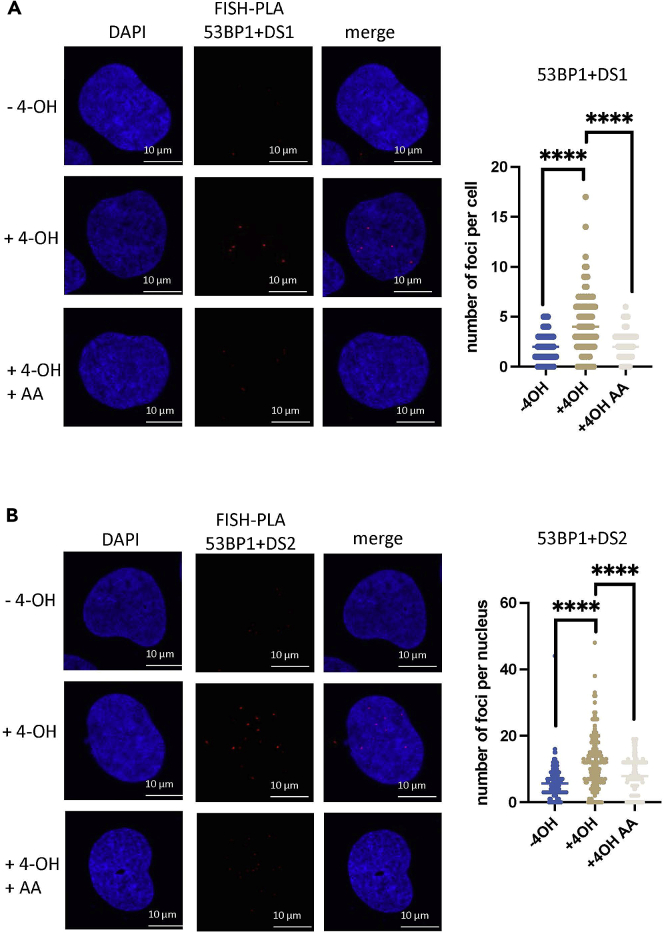
FISH-PLA showing 53BP1 proximity to nascent RNA at DSBs (A) Left, FISH-PLA of 53BP1 and DS1 probes, with and without 4-OHT (500 nM, 4 h) or α-Amanitin (2 μg/mL, 24 h). Right, quantification of right, (mean ± SD, n = 2), ∗∗∗∗p < 0.0001. (B) Left, FISH-PLA of 53BP1 and DS2 probes, with and without 4-OHT (500 nM, 4 h) or α-Amanitin (2 μg/mL, 24 h). Right, quantification of right, (mean ± SD, n = 2), ∗∗∗∗p < 0.0001. Images and quantification shown here were taken from^1^ and adapted for the purposes of this protocol.

**Figure 5:**
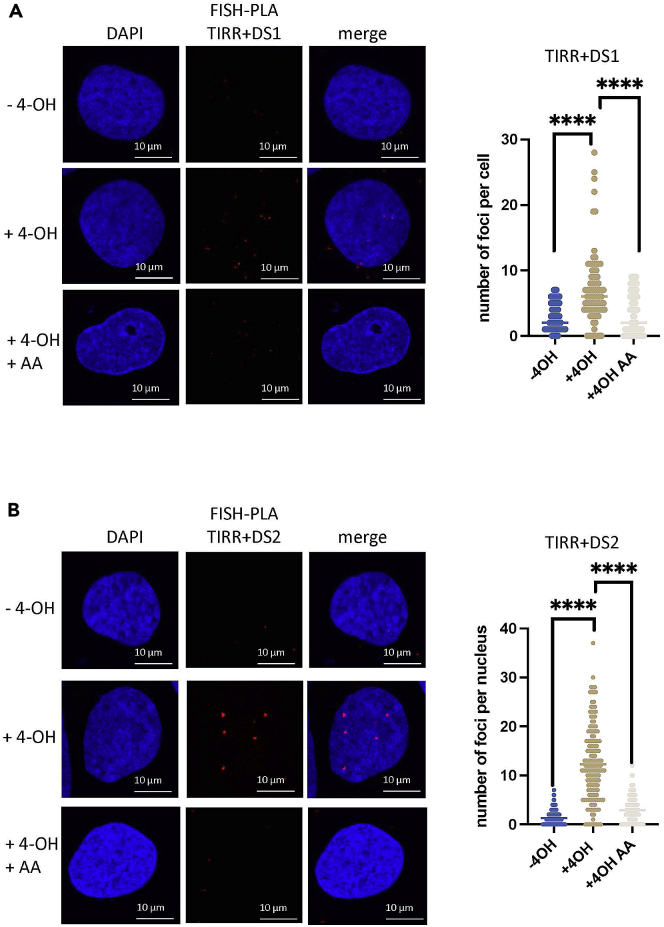
FISH-PLA showing TIRR proximity to nascent RNA at DSBs (A) Left, FISH-PLA of TIRR and DS1 probes, with and without 4-OHT (500 nM, 4 h) or α-Amanitin (2 μg/mL, 24 h). Right, quantification of right, (mean ± SD, n = 2), ∗∗∗∗p < 0.0001. (B) Left, FISH-PLA of TIRR and DS2 probes, with and without 4-OHT (500 nM, 4 h) or α-Amanitin (2 μg/mL, 24 h). Right, quantification of right, (mean ± SD, n = 2), ∗∗∗∗p < 0.0001. Images and quantification shown here were taken from^1^ and adapted for the purposes of this protocol.

For examples, see Figures 4 and 5 for FISH-PLA on 53BP1 and TIRR at DS1 and DS2.

**CRITICAL:** Saved images should maintain the C = 0 and C = 1 terms in the file name, as this is important for the image processing in CellProfiler software.

### Analysis of FISH-PLA images with the CellProfiler software

**Timing: variable from 2–24 h**

37. Download the Speckle Counter Pipeline (cellprofiler.org/examples#speckle-counting) and open the pipeline with the CellProfiler Software (File/Import/Pipeline from File).

***Note:*** The Speckle Counter Pipeline consists of several modules, each module is responsible for a specific image-processing step in the pipeline.

**CRITICAL:** The Speckle Counter pipeline should be optimized on a few images, ideally on positive and negative control images. The optimization step should evaluate the relationship between the foci and the nuclei, resulting in the automated identification of the number of foci per nucleus.

38. Cellprofiler settings can be modified by selecting specific input modules at the top left of the CellProfiler window (i.e., Images and Name And Types), as described below.
39. Select the Images module and upload the images to be analyzed (e.g sample_name.oib – C = 0 and sample_name.oib – C = 1) by dragging and dropping the files into the Images tab.
40. Select the NameAndTypes module and select “Images matching rules” next to the “assign a name to” section. Next to the “Select the rule criteria” and “File/Does/Contain” insert the value “C = 0”. In the box “Name to assign these images” insert “DAPI”. Select “Color image” or “Grayscale image” in the “image type” section, depending on the saved file after the Image J processing.
41. Repeat for the foci images, setting “C = 1” in the “Select the rule criteria”/“File/Does/Contain” and assign the name “FISHPLA”
42. Click “Update” at the bottom of the window to list the selected files.

***Note:*** this module assigns a name to each image, by which the following modules will refer to it.

43. Set the following settings in the downstream analysis modules (i.e., Identify Primary Objects, Mask Image, Measure Object Intensity, Relate Objects):
44. In the “Identify Primary Objects” section, select the input image value as “DAPI” for the identification of the nuclei and name the primary objects to be identified as “Nuclei”. Maintain the typical diameter of objects, in pixel units (Min, Max) within the range 30–200.
45. Select the input image as “FISHPLA” in the Mask Image and name the output image as “MaskedFoci”
46. In the second “Identify Primary Objects” section, select the input image value as “MaskedFoci” for the identification of the foci using per-object thresholding, and name the primary objects to be identified as “Foci”. Set the appropriate pixel units based on the size of the foci generated (usually between 4–35).
47. In the “Relate Objects” module identify the “Parent Objects” as Nuclei and the “Child Objects” as Foci.

**Figure 6:**
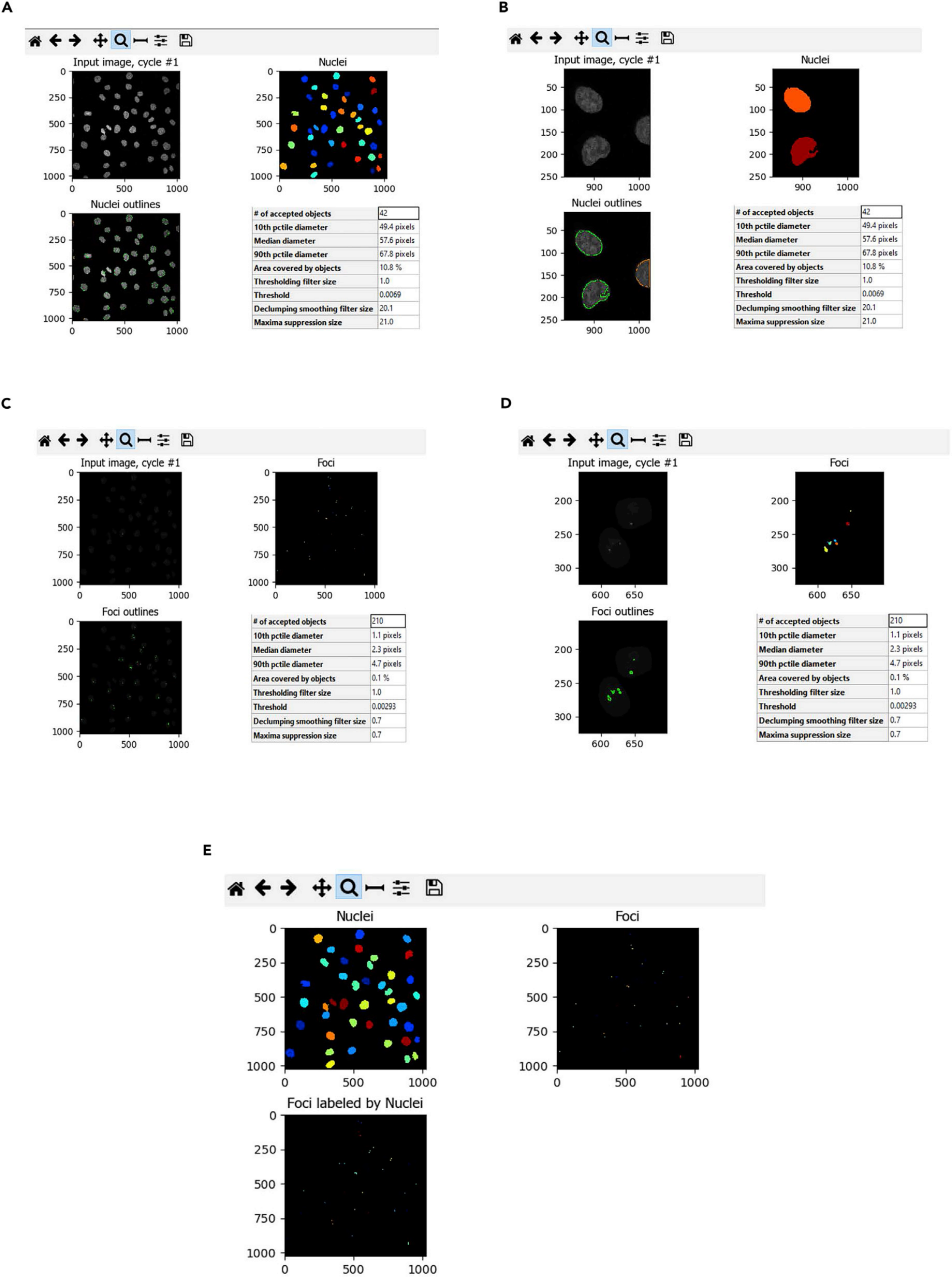
Screenshots of the CellProfiler output for the identification of the primary objects (A) Identification of the Nuclei (green circles). (B) Zoomed area from (A). (C) Identification of the Foci (green spots). (D) Zoomed area from (C). (E) Definition of the related objects (Nuclei + Foci).

For examples of the CellProfiler outputs see Figure 6.

***Note:*** To visualise the results of each module, select “Start Test Mode” located in the menu bar at bottom of the CellProfiler window. Pause and play icons will appear next to the different analysis modules. Select “Step” to visualize the outputs from each of the analysis modules (Figure 4).

48. Finally in the section “Export to Spreadsheet”, select the output folder and plot the Children Foci Count values present in the excel file “Nuclei” using GraphPad Prism software.

**CRITICAL:** For the accurate identification of both the nuclei and foci, some manual optimization in the input analysis settings may be required. Additional settings such as the threshold strategy, thresholding method, method to distinguish clumped objects, and method to draw dividing lines between clumped objects, can be optimised. In order to evaluate the results of the chosen settings, Test Mode can be used.

## Expected outcomes

Roles of RNA-binding proteins in the DNA Damage Response (DDR) have been described. Moreover, different RNA species ranging from short non-coding RNAs (i.e., microRNAs (miRNAs), long non-coding RNAs (lncRNAs), DNA damage-dependent small RNAs (DDRNAs)), to long damage-induced and transcription-related RNAs (i.e., damage-responsive transcripts (DARTs), and damage-induced lncRNA (dilincRNAs)) have also been described as crucial players in RNA-dependent DDR.^3^ Traditional crosslinking-based and RNA-tagged approaches (e.g., crosslinked immunoprecipitation (CLIP) and RNA-protein interaction detection (RaPID)) have been successfully employed to identify and describe novel RNA-protein interactions. However, these techniques have several limitations including significant background noise due to a high level of non-specific RNAs, subcellular mislocalization of tagged RNAs, and sensitivity issues for low expressed RNAs.^4^

Our FISH-PLA approach is a sensitive proximity ligation-based method for the proximal detection of sequence-specific RNA molecules and a protein of interest, overcoming some limitations associated with well-established assays for the detection RNA-protein interactions. The DNA probes used in this approach are a direct substrate for the PLA ligation step; this allows the detection of low abundant and transient RNA molecules, such as those produced upon DNA damage at DSBs.

We designed the DNA probes with strong and specific complementarity with the RNA of interest (50 nucleotides), with the addition of a linker of 41 deoxyadenosines that allows not only an efficient T4-mediated ligation during the PLA ligation step, but also ensures the detection of RNA-protein proximity within the canonical 40 nm PLA proximity range. The probes also contain a 22 nucleotide sequence adapter important for the ligation step.

**Figure 7:**
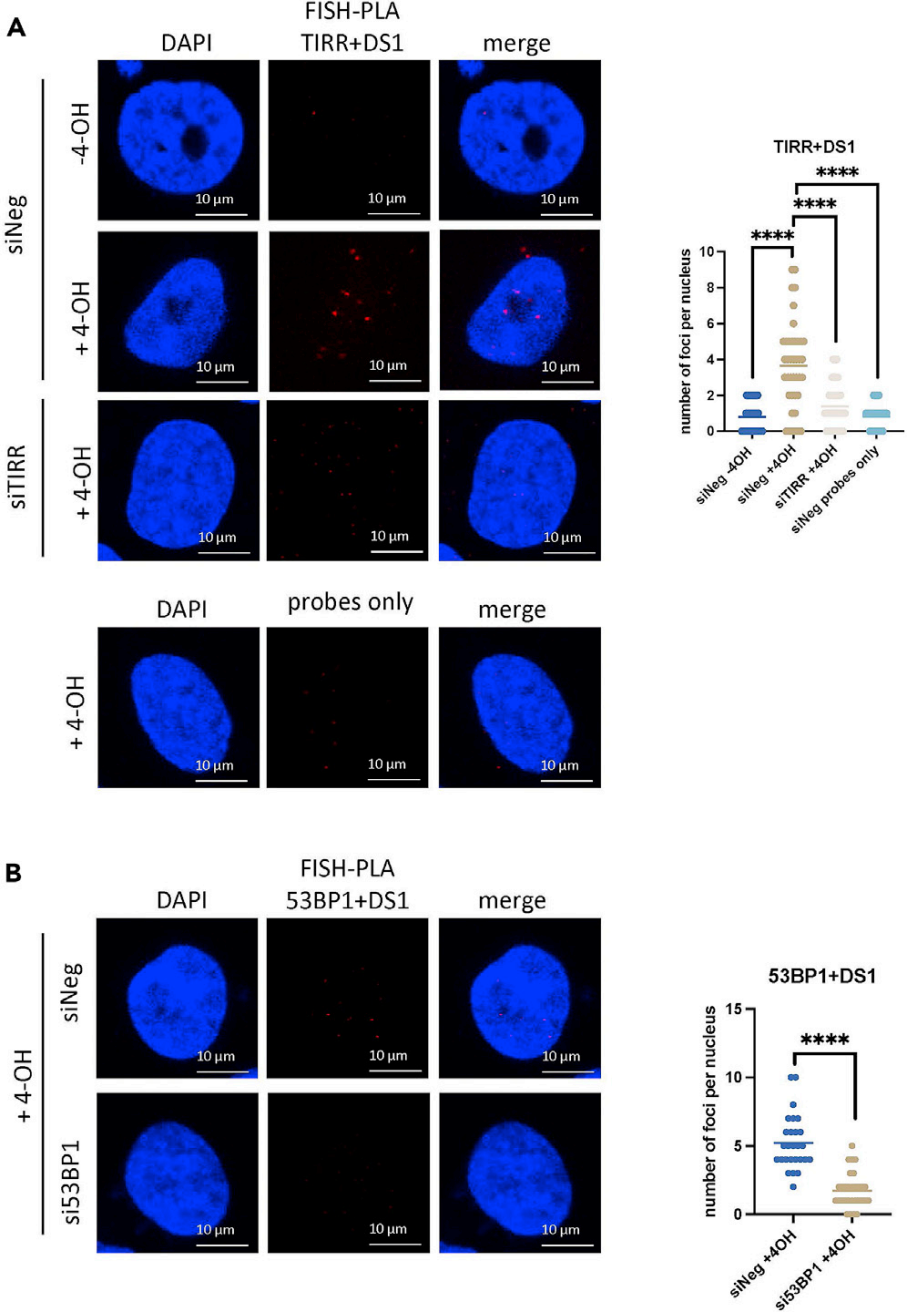
FISH-PLA showing TIRR and 53BP1 proximity to RNA produced at DS1, with siRNA controls and a probe-only control (A) TIRR and DS1, with siNeg and siTIRR, with 4-OHT. A probes-only control is shown (below). ∗∗∗∗p < 0.0001. (B) 53BP1 at DS1 with siNeg and si53BP1, with 4-OHT. ∗∗∗∗p < 0.0001.

Furthermore, to reduce the chances of false-positive results, we included some experimental controls such as: the addition of an α-amanitin incubation step that reduces the presence of the *de novo* transcribed RNA molecules, the detection of the RNA-protein interaction at two different DSB loci (DS1 and DS2), the employment of a primary antibody against a well-known repair protein 53BP1, which has previously been shown to interact with *de novo* transcribed RNA,^5^ and the use of siRNA knockdown and probe-only controls (Figure 7). It is also possible to use different experimental controls, such as scrambled DNA oligonucleotide probes.

This protocol could be used in a wide range of applications and can be extended to the detection and the validation of other RNA-protein interactions.

## Quantification and statistical analysis

RNA-protein interactions were visualized using Fiji Image J software^6^ and analyzed using CellProfiler™ software^7^ with the Speckle Counter pipeline. Statistical analysis was performed using GraphPad Prism 9 and the Mann Whitney test was performed.

## Limitations

In the canonical PLA assay, DNA probes covalently linked to the secondary antibodies with specific sequences are important for the ligation step. The PLA minus probe consists of a secondary antibody linked to a DNA moiety of 10 adenosines, and a specific DNA sequence (5′ GAC GCT AAT AGT TAA GAC GCT T) with three 3′-end 2′-O-methyl-RNA residues, while the PLA plus probe bears a DNA probe sequence characterized by a 10 adenosine linker and a DNA sequence 5′ TAT GAC AGA ACT AGA CAC TCT T. The 2′-O-methyl-RNA residues are necessary to decrease the T4 DNA ligase-mediated ligation efficiency (by about 50%) to avoid spurious ligation resulting in false positive artefacts.

In this study we decided to use DNA probes bearing the specific sequence of the PLA plus probes, due to synthesis issues with long 108-mer DNA/RNA molecules.

Nevertheless, this approach overcomes several issues of oligonucleotide-antibody PLA-based methods. For example, DNA probes are more stable compared to RNA probes and are not degraded by ubiquitous RNAses. Moreover, due to the higher thermodynamic stability of RNA:DNA hybrids over DNA:DNA duplexes, it is possible to ensure specific RNA hybridization through the use of sequential washes with incremental reduced salt concentration that enhance the hybridization stringency and RNA target recognition.

## Troubleshooting

### Problem 1

Unspecific FISH-PLA foci.

This may be related to high DNA probe concentrations and/or inappropriate antibody concentration.

### Potential solution

• Lower the DNA probe/antibody concentration and run experiment controls using DNA probes only and antibody only to check for non-specific signals.
• Titrate the DNA probe concentration by performing FISH-PLA experiments using α-amanitin or Triptolide as controls.
• Titrate the optimal antibody concentration by performing IF experiments with knockdowns as a control.
• More stringent wash conditions should be considered by increasing the washing time and/or by adding more washing steps.

### Problem 2

Low signal-to-noise ratio.

The source of this issue may depend on several steps:

• Fixation and permeabilization time.
• Drying out of the coverslips during the Duolink PLA washing.

### Potential solution

• Modify the PFA fixation time.
• Keep the coverslip moist during the Duolink PLA washing.
• Add a pre-extraction step before fixation.

### Problem 3

No PLA foci.

The absence of PLA signal may be caused by numerous reasons including:

• No 4-OHT-dependent DSB induction.
• Degradation of the target RNA.
• A low concentration of or poor quality DNA probes.
• Overconfluent cells at the time of the FISH-PLA experiment.

### Potential solution

• Check for efficient 4-OHT-mediated DSB induction by performing an IF experiment using the γH2A.X antibody.
• Use RNAse-free reagents and consumables.
• Make fresh DNA probes avoiding freeze-thaw cycles.
• Seed cells at a lower density.

### Problem 4

Poor correlation between the imaged PLA foci and PLA foci numbers by CellProfiler automated counting.

### Potential solution

• To avoid inaccurate signal measurement, advance settings in the CellProfiler analysis modules can be adjusted.
• Run the Test Mode option to manually analyze the primary objects segmentation (Nuclei and Foci) and to verify the assigned count numbers of each object.

## Resource availability

### Lead contact

Further information and requests for resources and reagents should be directed to and will be fulfilled by the lead contact, Monika Gullerova, monika.gullerova@path.ox.ac.uk.

### Materials availability

Most materials required are commercially available.

## Data Availability

This protocol used CellProfiler software and code Speckle counter pipeline, both are available at www.cellprofiler.org.
